# Mapping the rapid expansion of India’s medical education sector: planning for the future

**DOI:** 10.1186/s12909-014-0266-1

**Published:** 2014-12-17

**Authors:** Yogesh Sabde, Vishal Diwan, Ayesha De Costa, Vijay K Mahadik

**Affiliations:** R.D. Gardi Medical College, Ujjain, India

**Keywords:** Geographic information system (GIS), Private medical schools, Access, India

## Abstract

**Background:**

India has witnessed rapid growth in its number of medical schools over the last few decades, particularly in recent years. One dominant feature of this growth has been expansion in the private medical education sector. At this point it is relevant to trace historically and geographically the changing role of public and private sectors in Indian medical education system.

**Methods:**

The information on medical schools and sociodemographic indicators at provincial, district and sub-district (taluks) level were retrieved from available online databases. A digital map of medical schools was plotted on a geo-referenced map of India. The growth of medical schools in public and private sectors was tracked over last seven decades using line diagrams and thematic maps. The growth of medical schools in context of geographic distribution and access across the poorer and relatively richer provinces as well as the country’s districts and taluks was explored using geographic information system. Finally candidate geographic areas, identified for intervention from equity perspective were plotted on the map of India.

**Results:**

The study presents findings of 355 medical schools in India that enrolled 44250 students in 2012. Private sector owned 195 (54.9%) schools and enrolled 24205 (54.7%) students in the same year. The 18 poorly performing provinces (population 620 million, 51.3%) had only 94 (26.5%) medical schools. The presence of the private sector was significantly lower in poorly performing provinces where it owned 38 (40.4%) medical schools as compared to 157 (60.2%) schools in better performing provinces. The distances to medical schools from taluks in poorly performing provinces were longer [median 65.1 kilometres (km)] than from taluks in better performing provinces (median 41.2 km). Taluks farthest from a medical school were, situated in economically poorer districts with poor health indicators, a lower standard of living index and low levels of urbanization.

**Conclusions:**

The distribution of medical schools in India is skewed in the favour of areas (provinces, districts and taluks) with better indicators of health, urbanization, standards of living and economic prosperity. This particular distribution was most evident in the case of private sector schools set up in recent decades.

## Background

Medical schools in India are teaching institutions offering a five-and-a-half year medical education leading to a university qualification: a medical bachelor and bachelor of surgery (MBBS) in allopathic (modern western) medicine. Each of these institutions is attached to a large tertiary care hospital. Education in western medicine was formally introduced into India in 1835 when the British established Madras Medical School. Soon, in 1840, the Portuguese established a medical school in Goa. University-affiliated medical education became the norm after the 1850s with the opening of the first three Indian Universities in Chennai, Calcutta and Mumbai [1]. At the time of independence in 1948, India had 23 medical schools that trained medical doctors in western medicine. All except one in Vellore were owned by the government, and together, they produced not more than 4000 medical graduates a year [2,3]. The number of medical schools (and graduates) in India has grown rapidly in the six post-independence decades; the country now has the largest medical education system in the world [4]. The growing role of the private sector has been the most dominant feature of the medical education system over these decades, especially the last two [4,5]. India stands at the top of a list of countries with the largest numbers of privately-owned medical schools, followed by the United States [4,6,7].

Because they produce the necessary human resources to staff the health system, medical schools are a vital component of any health care system. In addition, medical schools have the potential to attract qualified medical teaching faculty and paramedical staff to practice, thus providing services where they are located [8]. They also create an academic environment with the potential to conduct scientific research in medical and allied sciences, all while supporting the routine health care system by providing training and continuing medical education [9]. Medical schools provide the surrounding population with greater access to physicians and specialized care beyond levels than might be available in the routine health system [10,11]. In India, establishment of a medical school implies the establishment of a large tertiary care hospital in the area, often with hundreds of beds in different medical and surgical specialties. The outreach services established by the medical schools complement the routine health system as they offer specialist care to the local population [12]. Some medical schools even offer services at subsidized costs. Medical schools, with their large number of residential staff and students, act as economic hubs offering employment opportunities to the local population [13]. Therefore, as academic institutions connected to large hospitals, medical schools have the potential to influence the local health care system, the health of the local population and the well-being of the local economy.

Given the role that medical schools play in supporting the health care and general development of the local community, it is important to ensure that all regions, particularly underserved ones, benefit from the opportunities created by hosting medical schools. Our study builds on earlier work conducted by Mahal et al. [14], which clearly shows a concentration of medical schools in economically better-off provinces. Our study extends the previous literature, which studied this situation until 2004. Much has changed since then as there has been rapid expansion in the country’s medical education sector. There remain high interprovincial inequalities in India, with a positive correlation between provincial GDP and density of health care providers, such as doctors, dentists, nurses and midwives [15]. The country also suffers from marked rural urban disparity: the number of allopathic doctors in urban areas is four times higher than in rural areas [16]. As a result, India’s National Rural Health Mission (NRHM) plans to further expand the medical education system in order to cater to the country’s health manpower needs [17]. The government of India has recently allocated substantial resources towards expanding medical education [18]. At this point in time, it is relevant to historically trace and map the development of India’s medical education since the country’s independence. India has the world’s largest medical education system, making it important to trace the changing role of the public and private sectors over time, particularly in the last decade. Further, this study includes visualization of all of India’s sanctioned medical schools’ geographic locations until 2012. We also describe the pattern of geographic distribution of medical schools while taking into consideration existing health care indicators as well as socio-economic indicators of development at provincial, district and sub-district levels (taluks). This paper supports planning by identifying districts that could be optimal locations for future medical schools.

The main objectives of this paper are as follows: (i) to describe the current geographic distribution of medical schools (public and private) across India, (ii) to trace the growth of the medical education sector in the country since independence and (iii) to study the relationship between the location of medical schools in India and the development indicators of the areas in which they are situated.

## Methods

### Study setting

India is a union of 28 provinces with 833 million (68.8% of 1210 million) people living in rural areas [19]. India’s provinces have widely varying socio-economic and health indicators. Provinces in India are divided into administrative units called districts, each with a population of between 0.5-5 million (1.5 million on average). According to official records, there are 640 districts in India that show wide variations in health and economic indicators [19-22]. Districts are further subdivided into administrative blocks (taluks) which represent smaller population subgroups (populations of 100,000 to 500,000) in the districts. In this study, we have used the term ‘taluk’ for these blocks.

### Data sources

#### Medical Council of India (MCI) database

This research relied on information of the medical schools teaching MBBS courses with regards to their location, ownership, year of inception and annual intake of students. This information was obtained from an online database maintained by the Medical Council of India (MCI), an autonomous body that sets standards to which medical schools must conform to ensure educational quality. MCI accredits schools using periodic ‘inspections’ to ensure that institutions conform. MCI’s recognition is mandatory for a medical school to legally train students. The present study included all medical schools in India providing medical education leading up to the MBBS qualification, as listed in the MCI database as of 30th January 2013 [2]. Medical schools were categorized as public or private based on their ownership. Private schools were those not owned by government but by private management [6].

#### Socio-demographic indicators

##### Provincial level

**Figure 1:**
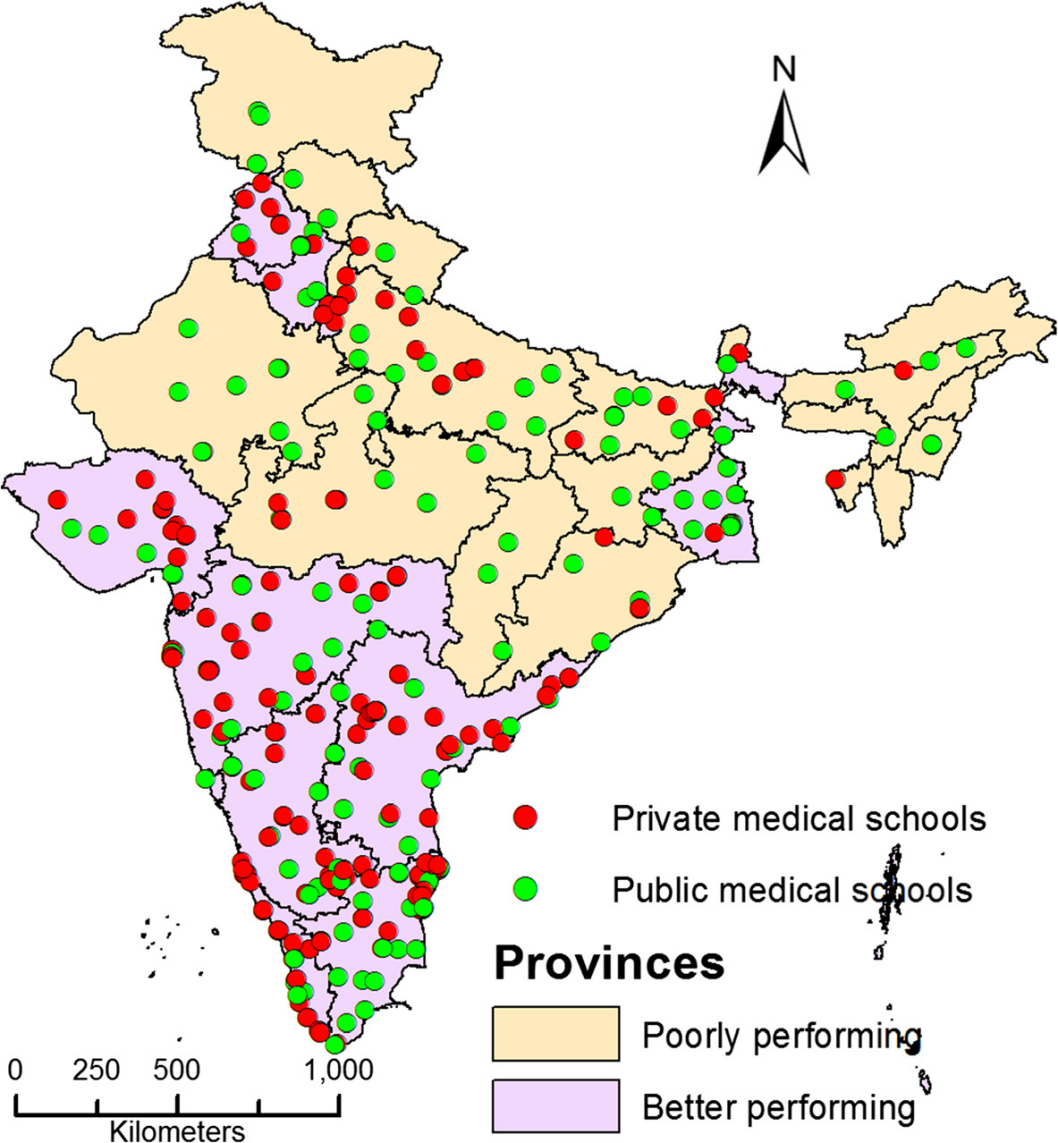
**Geographic distribution of medical schools in poorly and better performing provinces of India in 2012.**

We used the National Rural Health Mission (NRHM) classification of provinces. The NRHM classifies provinces based on socioeconomic and health care indicators (rather than wealth indicators). According to this classification, “high focus provinces” are those that have relatively poor indicators and require priority resource allocation to strengthen health systems [17]. Eighteen provinces, accounting for about 51% of India's population, have been designated ‘high focus provinces’ under India’s ongoing NRHM. According to national surveys conducted by the Indian government, these provinces have relatively poor socioeconomic indicators such as economic wealth and levels of poverty. These provinces have relatively higher maternal mortality ratios (MMRs), infant mortality rates (IMR) and lower life expectancy at birth than the national averages [23]. In this study, high focus provinces, as a group, are referred to as “poorly performing provinces” to differentiate them from other provinces, which are referred to as “better performing provinces” (Figure 1).

##### District level

Among the districts, this study used four distinct indicators of development - proportion of rural population, district’s ranking according to health care indicators, standard of living index and population below the poverty line - to represent levels of infrastructure, health, standard of living and economy, respectively. Details regarding district level development indicators are described below.

(i) *Proportion of rural population:* The proportion of rural population in each of the districts was considered, as reported by the official 2011 Census [19].
(ii) *District ranks:* The National Population Stabilization Fund is an autonomous society under the Ministry of Health and Family Welfare in India. It ranks the districts from a health perspective based on important maternal and child health indicators: i) percentage of women having three or more children, ii) contraceptive prevalence rate, iii) under five mortality rate and iv) antenatal coverage [20].
(iii) *Proportion of population with low standard of living index (low SOLI):* This measure reflects the population’s standard of living in a district. It is a composite index reflecting levels of housing and sanitation, economic status, ownership of assets and consumption of services, such as television and mobile phones. The 2007–2008 national district level household survey (DLHS) reported on the proportion of the population living with a low standard of living (SOLI) in each district [21].
(iv) *Poverty Status* – In India, a below poverty line (BPL) card is issued to financially disadvantaged households. The card is issued based on government evaluation of various parameters, including land ownership, type of house, sanitation, food security, household goods, literacy status and means of livelihood. Card holders are entitled to benefit from various welfare programs. The district-wide proportion of the BPL population was retrieved from the BPL Census 2011 from the government of India [22].

### Mapping

A digital map of India was purchased from the office of Survey of India (SOI) in Dehra Dun, India (License No. “BP11CDLA183”). The map outlined district boundaries as polygons and district head quarter towns as points. For security reasons, the boundaries and district head quarter points of 58 districts (that include the 22 districts of Jammu and Kashmir) were not available on this map. Therefore, the maps outlined 596 district boundaries with their head quarter points; these districts had 2254 sub-district level administrative blocks, which are called taluks. A digital map of the medical schools was subsequently prepared based on their location (addresses), as indicated in the MCI database and iconized on the map.

### Analysis

The proportions of medical schools in the public and private sectors were calculated for poorly performing and better performing provinces in the country. The number of medical schools and their annual intake capacity each decade were calculated over time between 1950 until 2010. The cumulative totals of these numbers (medical schools and their annual intake) were plotted using line diagrams.

The GIS tools used in the analysis were as follows [24]:

i. Distribution of medical schools and growth of medical education in poorly performing and better performing provinces of India:
  - *Thematic maps:* Maps that use symbols and colour coding systems to visualize quantitative data in a geographic map are called thematic maps [24]. The distribution of public and private medical schools across districts in India’s poorly performing and better performing provinces was shown using such a map. To study the growth of medical education facilities in India’s poorly performing and better performing provinces, the locations of medical schools in the public and private sectors were plotted for each decade since 1951.
  - Near analysis: To estimate the distance between adjacent medical schools, near analysis was used. The median near distances between the adjacent schools in poorly performing and better performing provinces were compared using independent samples Mann–Whitney U test.
ii. Access to medical schools for Taluks:
  - *Euclidean distances:* In this study, the straight line distance (Euclidean distance) to each taluk centroid (point) from the nearest medical school was used as an indicator of geographic access to the services offered by the medical school [24]. Euclidean distances were calculated in kilometres (km) using GIS tools. The Euclidean distances from a medical school for the taluks in poorly performing and better performing provinces were compared using a histogram.
  - *Ring buffer analysis:* Rings with radiuses of 50 km were plotted around the location of each medical school. The districts were classified in to two groups using the 50 km buffer zone: i) those with the district headquarter point within the buffer and ii) those with district headquarter point outside the buffer. The distribution (median and IQR) of the districts’ sociodemographic indicators were described for the districts within and outside 50 km buffer; these indicators included the proportion of rural population, ranking, proportion of population with low SOLI and proportion of people living below the poverty line.
iii. Identification of candidate geographic areas for intervention: Finally, based on the study’s variables, we screened districts that fulfilled all of the following criteria:
  - Euclidean distance of more than > 50 km
  - Population over 1 million
  - Rural population above 80%
  - District ranking lower than 300
  - Proportion of population with low SOLI above 20%
  - Proportion of BPL over 20%

The districts that fulfilled all of the above criteria were identified as “priority districts” for future medical school locations. The “priority districts” were plotted on the map of India using the background of poorly performing and better performing provinces for visualization.

Software used: PASW Statistics 18.0, STATA 12, ArcMap 10

### Ethical approval

The study did not use any human or animal subjects and/or tissue or field sampling. It uses publicly-available databases available on the websites of the following organizations: the Medical Council of India, the National Population Stabilization Fund, Census and DLHS. Because publicly-available databases were used in this analysis, no ethical approval was necessary. For mapping, we used the database obtained from the Survey of India; we were granted permission to use it in this research project.

## Results

### Distribution of medical schools in poorly performing and better performing provinces of India

In 2012, there were 355 medical schools in the country that enrolled 44250 students into physician training. The 18 poorly performing provinces with a population of 620 million (51.3%) had only 94 (26.5%) medical schools. Figure 1 shows the 2012 geographic distribution of 355 medical schools in India’s poorly performing and better performing provinces. The figure shows that private sector medical schools are largely located in the better performing provinces. This number as well as the proportion of privately-owned schools was lower in poorly performing provinces; there were 38 (40.4%) private schools in poorly performing provinces compared to 157 (60.2%) private schools in better performing provinces. The near analysis revealed the distance between two medical schools, which was significantly higher in poorly performing provinces than in better performing provinces (Median 43.8 and 3.7 km respectively, p value < 0.001 Mann–Whitney U test).

### Growth of medical education in poorly performing and better performing provinces

**Figure 2:**
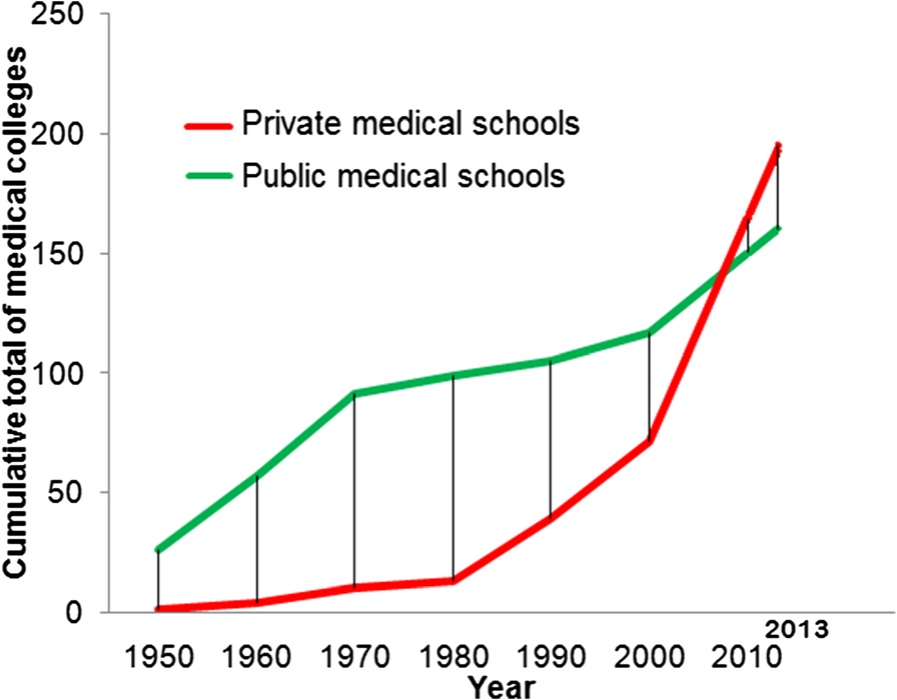
**Growth in the number of medical schools in India.**

**Figure 3:**
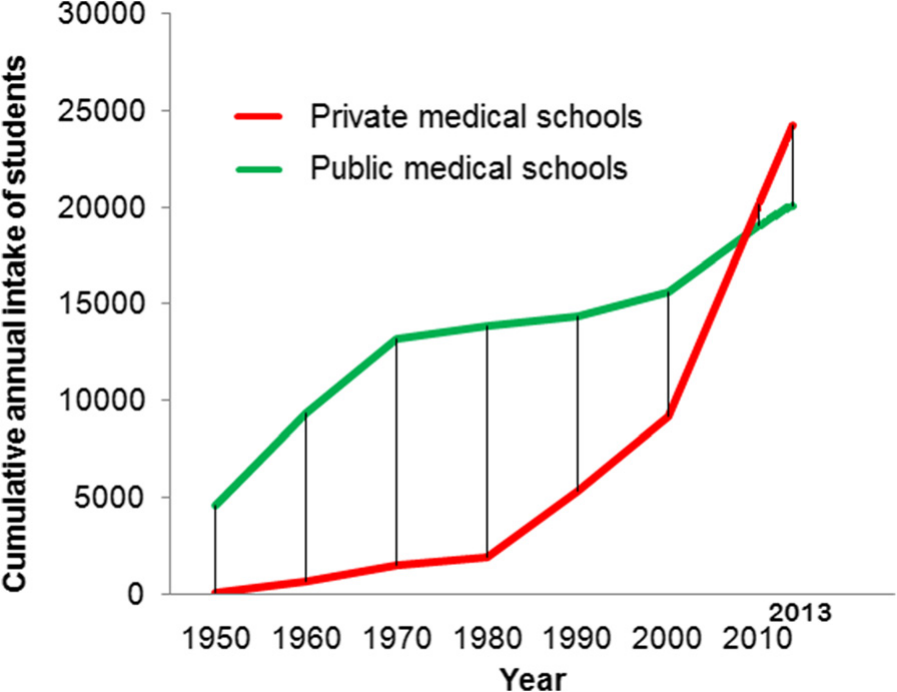
**Growth in the annual intake capacity of medical schools in India.**

**Figure 4:**
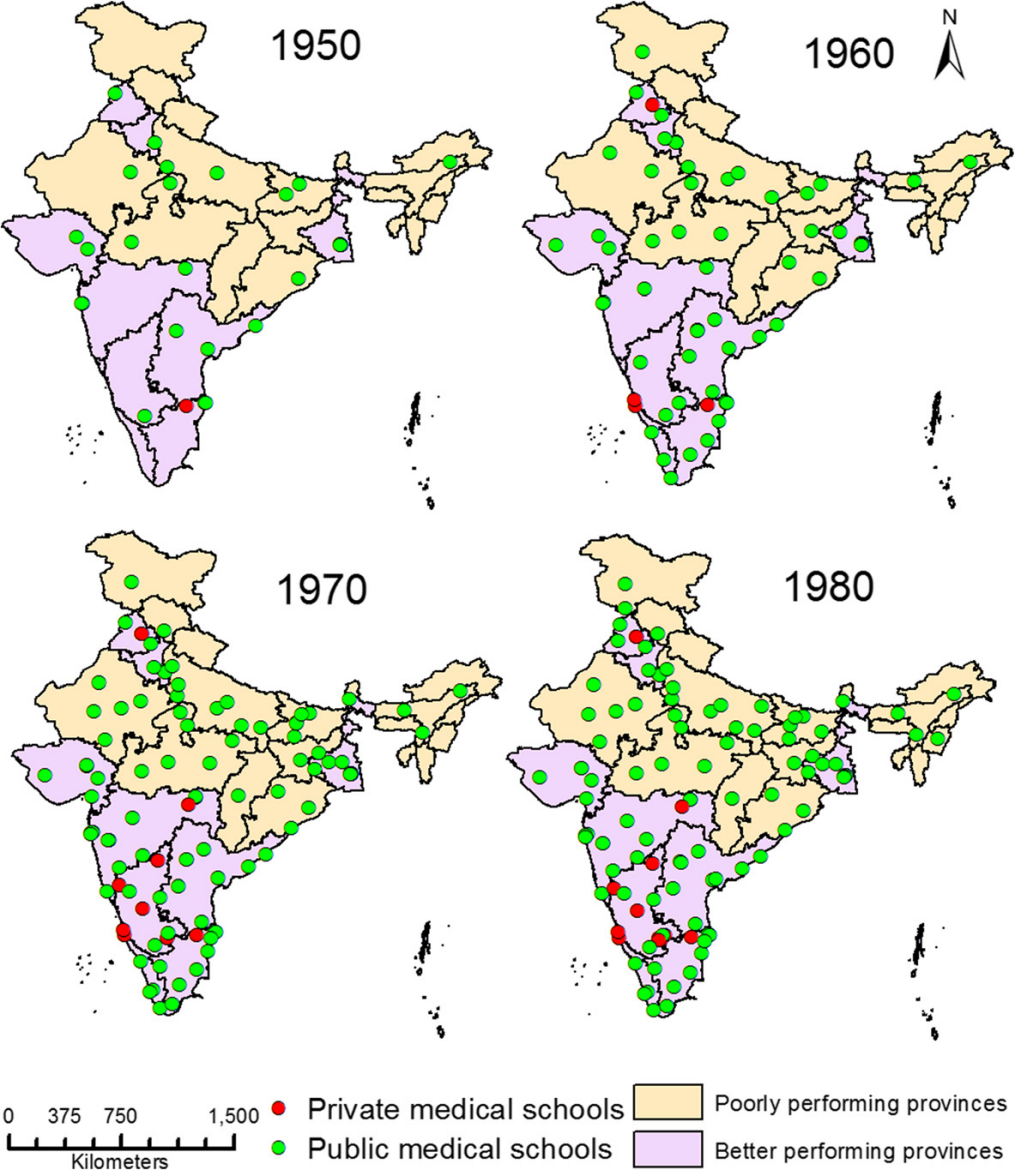
**Geographic distribution of medical schools in 1950 to 1980.**

**Figure 5:**
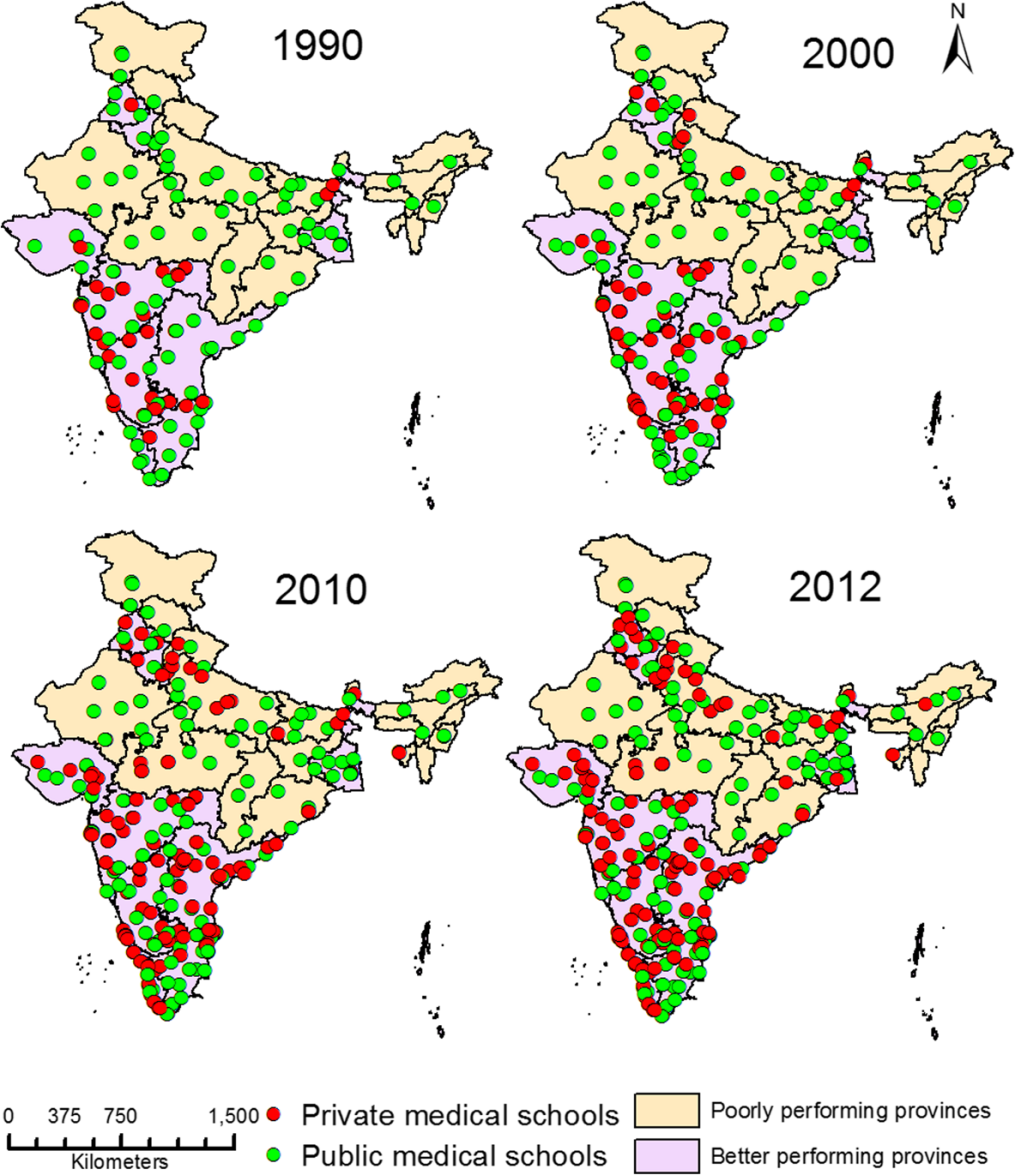
**Geographic distribution of medical schools in 1990 to 2012.**

Figures 2 and 3 show the growth in the number of medical schools and their annual intake capacity in the public and private sectors. The figures show that growth in the public sector reached a plateau following the 1970s. The private sector on the other hand, has grown exponentially since the 1980s, as seen in the figures. In India, the private medical education sector currently trains more students than the public sector (24205, 54.7%). Figures 4 and 5 show the geographic distribution of these schools in each decade from 1950 until 2012. These figures show that foci of private sector schools began in the south in the 1960s and then ‘spread’ to the north in the 1970s. The number of public sector schools also grew during this time. By the 1990s, there were many more private schools concentrated in the southern peninsula and the better performing northern provinces. After the 1990s, the public sector (no longer expanding) remained the major provider of medical education in the poorly performing provinces. However, the last decade has seen the establishment of private schools even in the poorly performing provinces.

### Access to medical schools for Taluks

**Figure 6:**
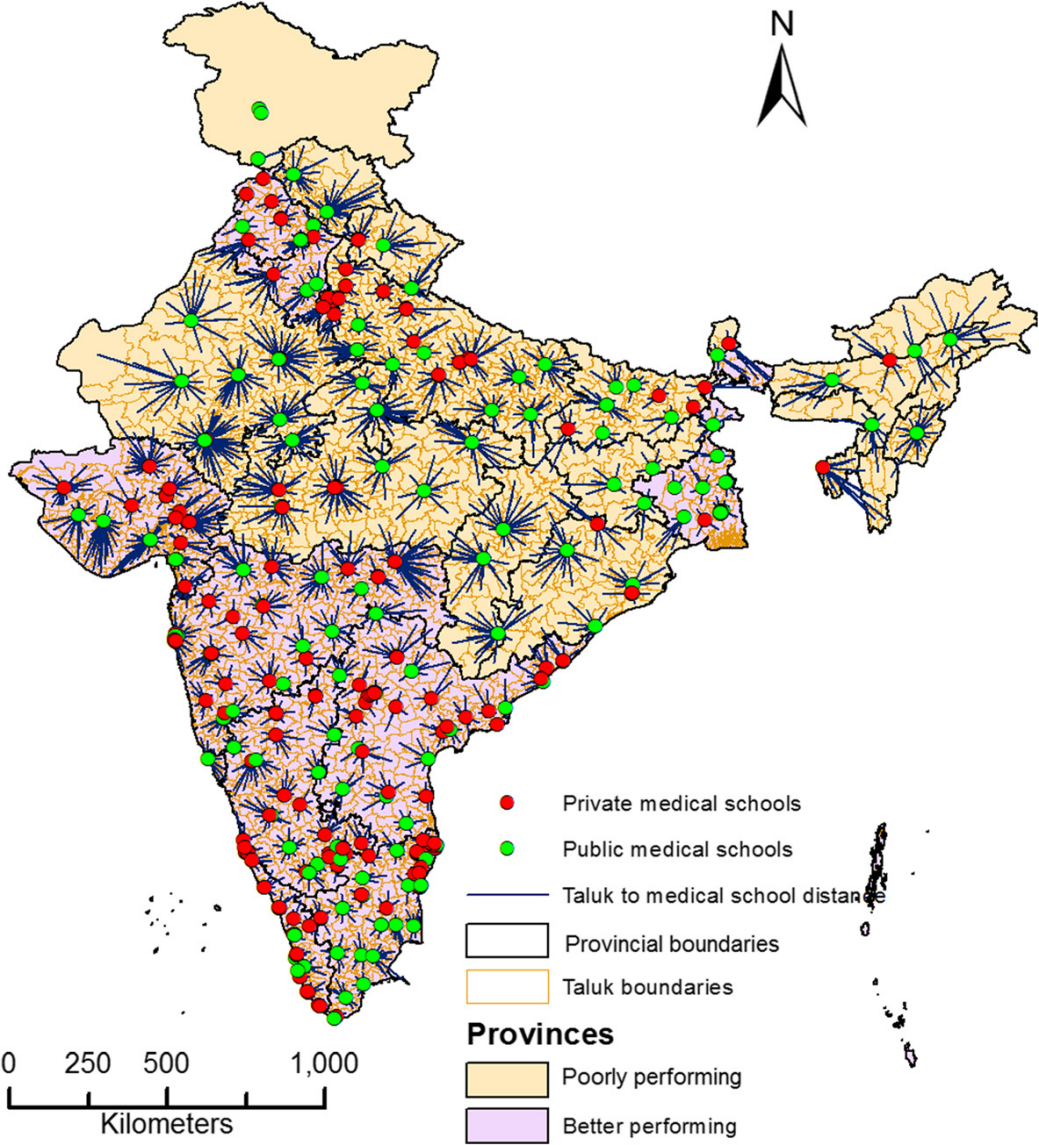
**Euclidean distance of taluks from medical school in India in 2012.**

**Figure 7:**
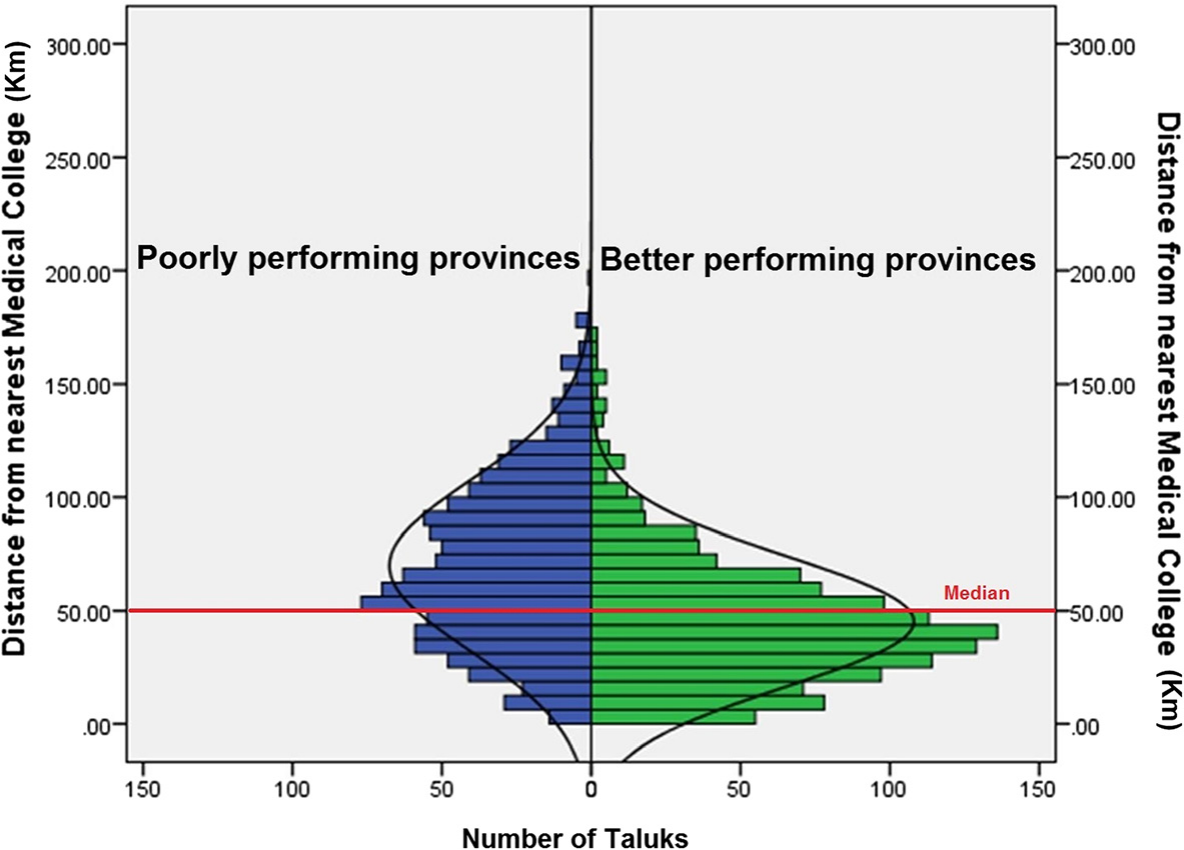
**Histogram showing Euclidean distances of taluks in poorly and better performing provinces.**

There were a total of 2254 taluks in India, of which 1101 (44.85%) were situated in poorly performing provinces. Figure 6 shows the Euclidean distances of the taluks from the nearest medical schools in poorly performing and better performing provinces. Medical schools in poorly performing provinces catered to more taluks over a wider area compared to medical schools in better performing provinces. The median distance of taluks from their nearest medical schools was 50.13 km (IQR 31.0 to 76.5 km). The median distance for the taluks in poorly performing and better performing provinces was 65.1 km (IQR 41.9 to 94.5 km) and 41.2 km (IQR 25.3 to 59.2 km), respectively. Figure 7 shows a histogram of taluks’ distances from nearest respective medical schools. The histogram was skewed to the left (towards zero) for the taluks in better performing provinces. Of the 1010 taluks in poorly performing provinces, 681 (67.4%) had distances to a medical school greater than the median distance of 50 km. The corresponding proportion in better performing provinces was 36.2%.

**Table 1:** **Distribution of development indicators among the districts within and outside 50 kilometres (km) distance from a medical school**

**SN**	**Development indicators**	**Districts (N = 582)**	
		**Within 50 km buffer N = 315 Median (IQR)**	**Outside 50 km buffer N = 267 Median (IQR)**
1	Rural population (%)	75.44 (61.75 – 84.76)	86.10 (79.32 – 92.11)
2	District rank (National Population Stabilization Fund)	185 (65 – 359)	330 (138 – 475)
3	Population with low Standard of Living Index (SOLI) (%)	7.2 (1.20 – 19.60)	20.1 (5.40 – 38.00)
4	Below poverty line population (%)	25 (14.30 – 37.70)	30 (17.20 – 43.70)

### Identification of geographic areas for intervention

**Figure 8:**
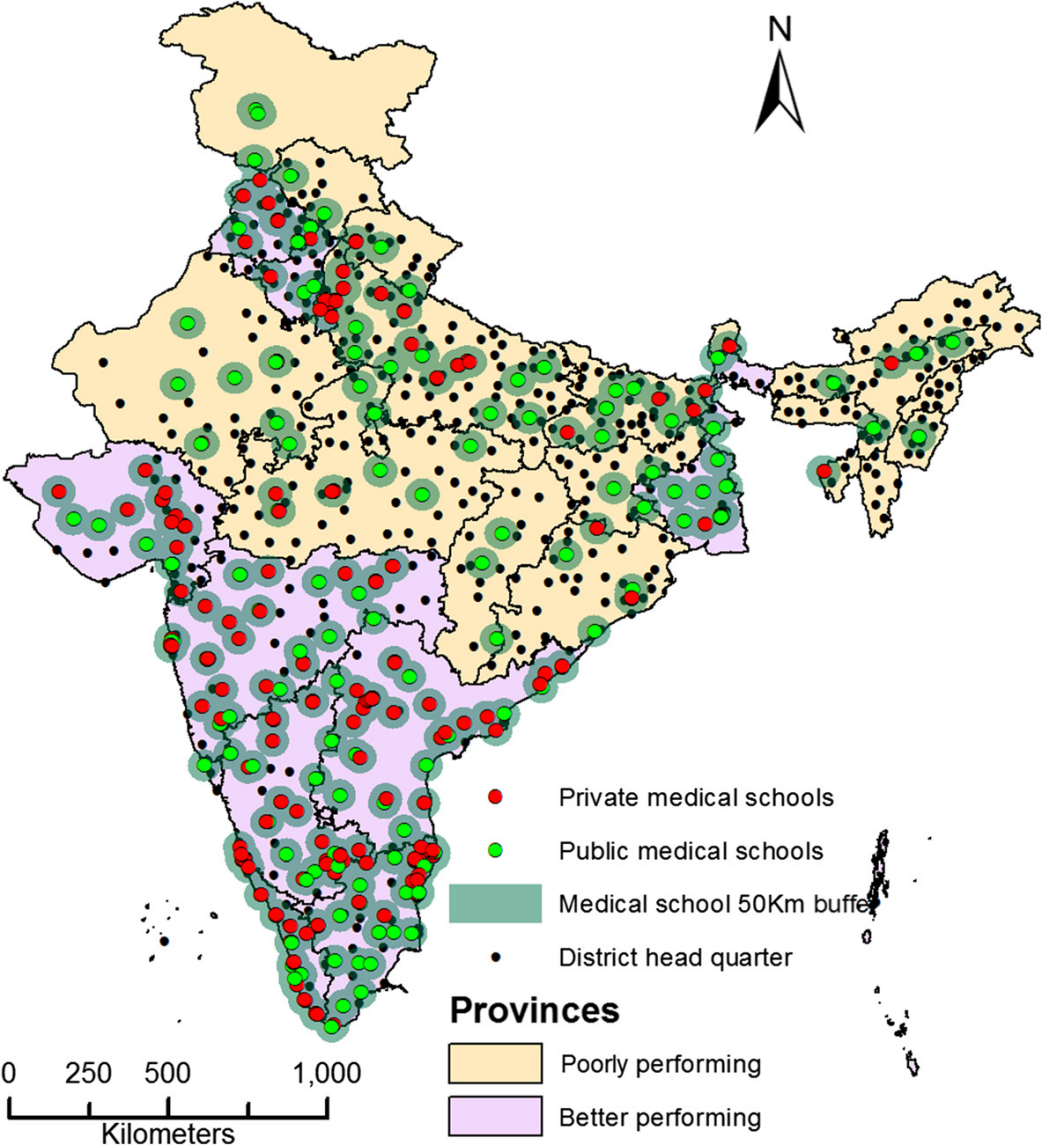
**District head quarters and 50 kilometres buffer zone of medical schools in India in 2012.**

**Figure 9:**
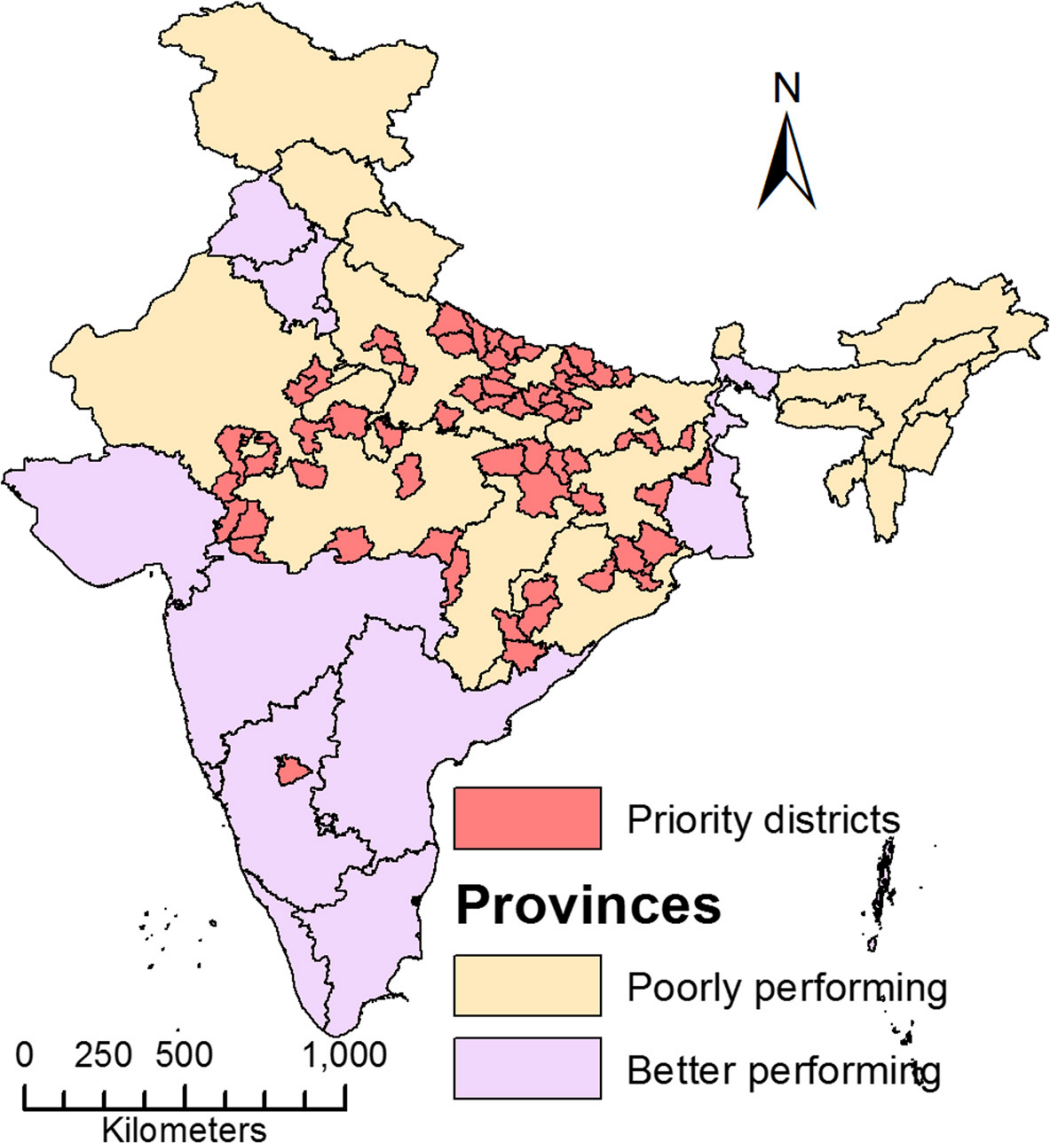
**Geographic distribution of identified clusters of districts which will benefit most with services of medical school.**

Figure 8 shows the geographic distribution of district headquarter points within and outside the 50 km buffer zone of medical schools. Of a total of 267 districts that were located >50 km away from a teaching hospital, 215 (80.5%) were in poorly performing provinces. Table 1 compares the distribution of development indicators among the districts within and outside the 50 km buffer from a medical school. Districts outside the 50 km buffer had higher proportions of rural individuals living below the poverty line with a low standard of living according to the index. Districts beyond the 50 km buffer had poorer ranks in terms of health indicators also. A total of 65 districts that fulfilled all the five specified criteria (described in methods) were identified as “priority districts” that would benefit most from the services of a medical school. Figure 9 shows the distribution of priority districts on a background of poorly performing and better performing provinces. Figure 9 shows that the “priority districts” are not randomly distributed; rather, they are clustered in certain geographic areas of the country (clusters of districts). The total population of these districts is 126 million (12.4%). 62 of these 65 districts were located in poorly performing provinces, which are currently high focus provinces under India’s NRHM.

## Discussion

In this study, we mapped 355 medical schools in the public and private sectors. The growth of the medical education system has been dominated by the private sector, particularly in the last few decades. In 2012, the private sector owned 195 (54.9%) schools and enrolled 24205 students (54.7%). The private sector medical schools were concentrated in the country’s better performing provinces. The distance from the nearest medical school was longer for the taluks in poorly performing provinces and districts compared to those located in better performing provinces and districts.

### Growth of medical education globally

The number of medical schools has increased in all parts of the world due to increasing populations, advances in technologies and increasing lifespans [6]. Asia (the most populous region) has witnessed the largest growth; it has 44% of the total medical schools in the world. Six of the top ten countries with the highest number of medical schools in the world are in Asia, including India. India has a large medical education system, producing nearly 45000 trained physicians annually [2,6,7]. There are many reasons for this trend in Asia, an important one being the need for trained physicians to serve the growing population. The demand for physicians has increased further due to medical tourism and overseas employment opportunities for medical professionals from developing countries. Today, India is the biggest source of foreign-trained physicians to the United Kingdom, the United States, Canada, Australia, the United Arab Emirates and New Zealand. Indian physicians are the largest emigrant physician workforce in the world. Almost 60,000 emigrant Indian physicians practice in the aforementioned countries. At the same time, there is an increasing number of aspirants to the medical profession given the desirability of a medical education [4].

### Differential growth of medical education in poorly performing and better performing geographic areas

The findings of our study revealed that the proportion of medical schools in the 18 poorly performing provinces of India was much lower than in the better performing provinces. The distances between adjacent medical schools were longer in poorly performing provinces. As a result, the population subgroups represented by taluks in the poorly performing provinces were significantly farther from a medical school. The distances of the districts with poorer development indicators were also farther from a medical school. Available studies have documented differential growth in the number of medical schools in India’s economically richer and poorer provinces [3,14]. In 2006, Ajay Mahal showed that medical schools were concentrated in the wealthier provinces [14]. Another study in 2010 showed that only 11% of all medical schools were located in the northern and eastern provinces of Bihar, Orissa, West Bengal, Manipur, Assam and Tripura; meanwhile, 61% of all medical schools in country were located in the six southern provinces of Maharashtra, Karnataka, Kerala, Tamilnadu, Andhra Pradesh and Pondicherry [3]. In our study, the corresponding proportions in the aforementioned northern and southern provinces were similar: 11.8% and 56.3%, respectively, in 2012. In general, the southern provinces have had better socio-economic indicators compared to the northern provinces [3]. Another Indian report has suggested that the location of a medical school seems to be influenced by political will and the paying capacity of the community [25].

### Role of private sector in the growth of medical education

The decades following the 1980s witnessed exponential growth in the private sector of medical education. The number as well as the student intake capacity of private medical schools exceeded that of public medical schools in 2012. Privatization of medical education over the past several decades has substantially contributed to the growth of medical education in Asia. More than half of medical schools are private in India, Nepal, Bangladesh, Pakistan, Japan, South Korea, Taiwan, Philippines, Malaysia, Singapore, United Arab Emirates and Oman. Meanwhile, all of the schools in Yemen, Qatar and Bahrain are in the private sector [6]. A number of reasons have been documented for this exponential growth in the private sector of medical education [5]. Mullan reported high interest in emigration as one possible motive behind growth in the privatization of medical education even though private medical schools do not exclusively educate for emigration [26]. In addition, given the high desirability of medical education in these settings, a number of investors, including political representatives and business communities, saw this sector as a lucrative business opportunity [5]. It has been reported that establishing medical schools in the private sector provides large investor profits. As the law in India requires that medical schools be run by nonprofit bodies, a number of legal forms of organization, including ‘trusts’ and ‘societies’, were set up by interested parties to establish and run these schools [12]. Though such organizational forms imply ‘non-profit’ bodies, often the schools are eventually run as ‘for-profit’ undertakings without explicitly stating so [6]. The entry of the private sector into medical education has been beset with controversy; concerns have been raised about the possible dilution of standards in medical education as well as medical education becoming accessible only to the rich [3,14,25]. Amin et al. also questioned the ethics in the establishment of some of these schools [27], while Sood R highlighted the need to check for the growing commercialization of medical education [28].

### Why are investors more willing to start medical schools in better off districts and provinces?

The growth of private sector institutions occurred mainly in the better performing provinces and districts with better development indicators. Most private sector schools have to raise their own funds to meet the requirements for sustained investment. Their main resource streams come from student fees and patients attending the attached hospital [3,6]. The interest of investors is driven by the paying capacity of the population. Students and patients who can afford these charges are more likely to live in richer areas as compared to poorer ones. Secondly, this choice is facilitated by the availability of large numbers of qualified human resources to staff medical schools in better-off areas; it is easier to attract and retain qualified staff in developed areas as compared to poorer areas [25]. Thirdly, the richer provinces and districts have better infrastructure and are thus able to attract investors in private medical education to the area [3,14,25]. Thus far, existing regulations for starting new medical schools mainly focus on the infrastructural requirements, assets and financial capacities of the owners. When accrediting new medical schools, no consideration is given to the existing health services in the local geographic area [12,14,25]. With incentive drivers and regulations that disregard location, private medical schools are more likely to be situated in better-off provinces and districts, unless future expansion is planned for both public and private sector schools [3]. Though privatization has contributed substantially to the pool of medical schools, there seems to be no indication of planned growth in this sector in the Indian setting.

### Why should geographic areas without medical schools have them?

The districts in poorly performing provinces and districts are not only poorer in terms of economic indicators; they also have poorer health care indicators, such as higher maternal mortality ratios (MMRs) and higher infant mortality rates (IMR) than the better performing districts [23]. The districts’ poor development indicators (health care in particular) suggest a need to consolidate health care delivery, which can be strongly supported by the services of a medical school. Medical schools themselves act as important stakeholders in the health care delivery system, as they provide tertiary care services as well as outreach for primary care services in the defined geographic area [8-13]. Distance is a very important barrier in emergency conditions like trauma, maternal (particularly perinatal) and newborn care as well as emergencies with non-communicable diseases (NCDs). Many of these emergencies require care from a tertiary care centre. For example, the availability of blood and caesarean sections can be life-saving in the case of potential maternal deaths. Poorly performing provinces and districts suffer disproportionately from these problems; the capacity to provide such services is limited in existing tertiary care centres (i.e. public sector district hospitals and private nursing homes). A medical school has the possibility of offering specialized care and management in life-threatening situations, particularly in relation to maternal, neonatal and child health [29-33]. Given the potential of medical schools to influence local health care services as well as the local economy, it is pertinent to prioritize districts without any medical school when deciding on the location of future medical schools.

In this study, we have identified the poorest districts in terms of socio-economic and health indicators and prioritized them based on their access to medical schools and the size of their total population. These districts, with populations of over one million, had no medical schools within 50 km of their main towns. The selection criteria applied in this study were chosen from an equity perspective, giving priority to districts with a poorer population in terms of economics, health, standard of living and infrastructure. Figure 9 shows that most of the identified districts lie in geographically contiguous clusters. The majority of these “clusters of districts” were located in poorly performing provinces. The National Rural Health Mission of India plans to take steps to expand medical education in order to address the health manpower crisis in India. These steps include revision of the MCI regulations for setting up medical schools and increasing intake capacities. In the general budget of 2013–14, the government of India proposed to provide Rs. 47.27 billion for medical education, training and research [18]. We recommend consideration of the identified geographic clusters when locating the new medical schools in public sector. Given that the majority of these “clusters of districts” were located in poorly performing provinces, the establishment of medical schools at these locations will help locate healthcare services in high focus provinces under India’s national health mission. To attract private entrepreneurs, it is important to provide a rationalization of the norms regarding land area and infrastructure for a medical school, particularly in under-served and difficult areas [23]. However, it is also important to note that there are many other factors that determine the location of a medical school in a geographic area. These factors include geographic terrain, political will, human resource availability, infrastructure and clinical material. In some of these identified “district clusters” it might not be possible to start new medical schools given serious potential deficits in these other factors. Our conclusions have not taken into consideration these other factors; we have come to our conclusions using an equity-based perspective. Therefore, the identified “priority areas” challenged by other factors can be candidates for the proposed three-and-a-half year degree in modern medicine, the bachelor of rural medicine and surgery (BRMS) [16,34]. These courses are based on the experiences of provinces with difficult geographic areas like Chhattisgarh and Assam [34].

## Conclusion

The findings of the present study are important given that there are no studies describing the distribution of medical schools against district and sub-district level development indicators. We used a geographic visualization of the medical school distribution across the country and traced the growth of India’s medical education sector. This study described the access to medical school for various provinces, districts and taluks in India. The distances for the taluks in poorly performing districts and provinces were longer than the taluks in better performing provinces. Clearly, the rapid expansion of the country’s private sector for medical education, primarily in the better off provinces and districts, is the key determinant for the differential growth pattern. The rapid growth of medical education is expected to continue in the coming years; meanwhile, India’s National rural health mission provides an impetus for medical education. It is therefore important to plan the location of new medical schools, public and private, from an equity and organizational perspective. This study has identified “clusters of districts” that would benefit most from the services of medical schools. It is recommended that the identified “clusters of districts” be given priority for the establishment of future medical schools.
